# The role of human papillomavirus in head and neck cancer in Senegal

**DOI:** 10.1186/1750-9378-8-14

**Published:** 2013-04-17

**Authors:** Cathy Ndiaye, Laia Alemany, Yankhoba Diop, Nafissatou Ndiaye, Marie-Joseph Diémé, Sara Tous, Jo Ellen Klaustermeier, Maria Alejo, Xavier Castellsagué, F Xavier Bosch, Helen Trottier, Silvia de Sanjosé

**Affiliations:** 1 Department of Social and Preventive Medicine, Université de Montréal Sainte-Justine Hospital Research Center, 3175 Côte Sainte-Catherine, Room A-830, Montreal, QC H3T 1C5, Canada; 2 Unit of Infections and Cancer, Institut Català d'Oncologia, Barcelona, Spain; 3 CIBER Epidemiología y Salud Pública, CIBERESP, Barcelona, Spain; 4 Hôpital Principal de Dakar, Dakar, Senegal; 5 Université Cheikh Anta Diop, Dakar, Senegal; 6 Red Temàtica de Investigaciòn Cooperativa en Càncer, RTICC, Barcelona, Spain; 7 Sainte-Justine Hospital Research Center, Montreal, Canada

**Keywords:** Head and neck cancer, Human papillomavirus, Senegal, Sub-Saharan Africa

## Abstract

**Background:**

Exploring the presence and role of human papillomavirus (HPV) in head and neck cancer (HNC) is a necessary step to evaluate the potential impact of HPV prophylactic vaccines.

**Objective:**

To assess the prevalence and oncogenic role of HPV in HNC in Senegal.

**Methods:**

This is a multicenter cross-sectional study. Paraffin-embedded blocks of cases diagnosed with invasive HNC between 2002 and 2010 were collected from 4 pathology laboratories in Senegal. Presence of HPV DNA was determined by PCR and DEIA, and genotyping performed with LiPA_25_. Tubulin analysis was performed to assess DNA quality. HPV DNA-positive cases were tested for p16^INK4a^ expression.

**Findings:**

A total of 117 cases were included in the analysis: 71% were men, mean age was 52 years old (SD ±18.3), and 96% of cases were squamous cell carcinoma. Analysis was performed on 41 oral cavity tumors, 64 laryngeal tumors, 5 oropharyngeal tumors and 7 pharyngeal tumors. Only four cases (3.4%; 95% CI = 0.9%-8.5%) harbored HPV DNA. HPV types detected were HPV16, HPV35 and HPV45. However, among HPV-positive cases, none showed p16^INK4a^ overexpression.

**Conclusion:**

Our findings indicate that HPV DNA prevalence in HNC in Senegal is very low, suggesting that HPV is not a strong risk factor for these cancers. Additional larger studies are needed to confirm these findings and explore other potential risk factors specific to the region.

## Introduction

Head and neck cancer (HNC) is the eighth most common cancer worldwide with approximately 650,000 new cases and 350,000 deaths reported each year [1]. In addition to alcohol and tobacco consumption, the latest evaluation on the carcinogenicity of infections and cancer by the International Agency for Research on Cancer established human papillomavirus (HPV) as a carcinogen of the oral cavity and oropharynx [2].

In sub-Saharan Africa, knowledge on the role of HPV in HNC is very limited. Actually, 84% of the existing information on HPV and HNC is derived from studies in Europe and North America [3]. The aim of our research is to provide original data on the role of HPV in invasive HNC in Senegal.

## Material and methods

This is a cross-sectional study designed and coordinated by the Catalan Institute of Oncology in Barcelona and is part of a large international study on HPV in HNC. Four major centers in Dakar, Senegal, have provided consecutive cases diagnosed with HNC from 2002 to 2010: Centre Hospitalier Universitaire A. Le Dantec, Hôpital Principal de Dakar, Hôpital Général de Grand Yoff and the pathology laboratory at Cheikh Anta Diop University. For each specimen, after pathological confirmation of invasive diagnosis, a paraffin tissue section was treated with 250 μl of freshly prepared Proteinase K solution to extract DNA. SPF-10 polymerase chain reaction (PCR) was performed using 10 μl of a 1:10 dilution of the crude DNA extract in a final reaction volume of 50 μl. The amplified PCR products were tested using a probe hybridization step with a cocktail of conservative probes that can recognize around 54 mucosal HPV genotypes using a microtiter plate format for the detection of HPV DNA through a DNA enzyme immunoassay (DEIA) (produced by DDL, Voorburg, the Netherlands). Optical densities (OD450) were read on a microtiter plate reader and categorized as HPV DNA negative, positive, or borderline. After PCR, 10 μl of the DEIA HPV DNA positive amplimers were used to perform the reverse hybridization line probe assay (LiPA_25_) (version 1: produced by DDL, Voorburg, the Netherlands). The LiPA_25_ detects 25 high-risk and low-risk HPV types (6, 11, 16, 18, 31, 33, 34, 35, 39, 40, 42, 43, 44, 45, 51, 52, 53, 54, 56, 58, 59, 66, 68, 70, and 74). Additionally, amplification of the human tubulin gene was performed to determine DNA quality of DEIA negative samples. In HPV DNA-positive samples, p16^INK4a^ overexpression was evaluated using CINtec histology kit (clone E6H4, mtm Laboratories, Heidelberg, Germany) in order to verify the potential causal role of HPV in positive cases. Overall HPV detection percentages and 95% confidence intervals (95% CI) were estimated. Data analyses were performed with STATA 11.0. Ethical approval was obtained from the Ministry of Health of Senegal.

## Findings

**Figure 1:**
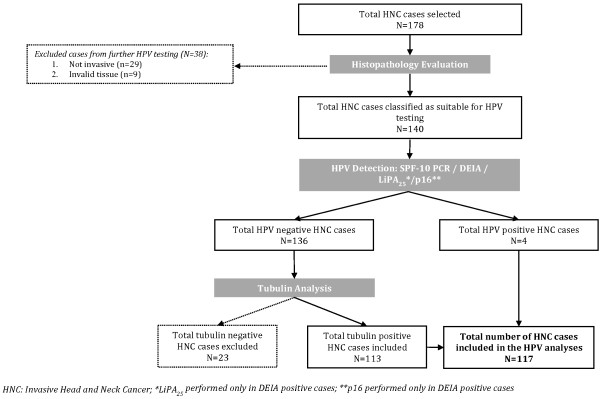
Algorithm of head and neck cancer cases included in the study.

Figure 1 displays the algorithm of sample selection for analysis. The final number of cases included in the study was 117: 41 oral cavity tumors, 64 laryngeal tumors (including 25 tumors whose site could not be discerned between hypopharynx and larynx), 5 oropharyngeal tumors and 7 pharyngeal tumors. Description of the cases is presented in Table 1. In this study, 70.9% of cases were men and mean age was 52 years old (SD ±18.3). Squamous cell carcinoma (SCC) represented 95.7% of cases.

**Table 1:** HPV DNA detection in head and neck cancer cases in Senegal by subjects’ characteristics

**Variables**	**Cases tested for HPV**		**HPV positive cases**	
	**N**	**%**	**N**	**% (95% CI)**
**Gender**				
Male	83	70.9	1	1.2 (0.0-6.5)
Female	34	29.1	3	8.8 (1.9-23.4)
**Age at diagnosis (in years)**				
≤ 39	28	23.9	1	3.6 (0.0-18.3)
40-49	16	13.7	1	6.2 (0.2-30.2)
50-59	19	16.2	0	0.0 (0.0-17.6)^b^
≥ 60	49	41.9	2	4.1 (0.5-14.0)
Missing	5	4.3	0	0.0 (0.0-52.2)^b^
**Period of diagnosis**				
2002 - 2008	53	45.3	3	5.7 (1.2-15.7)
2009 - 2010	64	54.7	1	1.6 (0.0-8.4)
**Histological type**				
Squamous cell carcinoma	112	95.7	4	3.6 (1.0-8.9)
Adenocarcinoma	1	0.9	0	0.0 (0.0-97.5)^b^
Other diagnoses^a^	4	3.4	0	0.0 (0.0-60.2)^b^
**Total**	**117**	**100**	**4**	**3.4 (0.9-8.5)**

^a^ Other diagnoses include 2 small cell neuroendocrine carcinomas and 2 adenoid cystic carcinomas.

^b^ One-sided, 97.5% confidence interval.

The overall HPV prevalence was 3.4%; 95% CI = 0.9%-8.5%. Table 2 shows the number of cases per subsite, the distribution of HPV genotypes and results for p16^INK4a^ expression. HPV DNA was detected in 4 carcinomas: one gingival SCC (HPV35) and three laryngeal SCC (2 HPV45 and 1 HPV16). However, none of these cases showed p16^INK4a^ overexpression: 3 cases showed no p16^INK4a^ staining and one HPV45 laryngeal cancer case showed focal staining of less than 25% of cells with low-moderate intensity.

**Table 2:** **HPV DNA, HPV types and p16**^INK4a^**detection in HPV positive cases by head and neck cancer sites and subsites**

**HNC sites and subsites**	**Cases tested for HPV**		**HPV DNA positive**		**HPV type**	**p16**^**INK4a **^**overexpression**
	**N**	**%**	**N**	**% (95% CI)**		
**Oral cavity**						
Lip	4	3.4	0	0.0		
Gingiva	5	4.3	1	20.0 (0.5-71.6)	HPV35 (N = 1)	Negative
Floor of the mouth	1	0.9	0	0.0		
Tongue	25	21.4	0	0.0		
Hard palate	3	2.6	0	0.0		
Palate unspecified	2	1.7	0	0.0		
Oral cavity unspecified	1	0.9	0	0.0		
**Larynx**						
Epiglottis	2	1.7	0	0.0		
Vocal cord	2	1.7	0	0.0		
					HPV45 (N = 2)	
Larynx unspecified	35	29.9	3	8.6 (1.8-23.1)	HPV16 (N = 1)	Negative
Hypopharynx or larynx	25	21.4	0	0.0		
**Oropharynx**						
Tonsil	1	0.9	0	0.0		
Oropharynx unspecified	4	3.4	0	0.0		
**Pharynx**						
Pharynx unspecified	1	0.9	0	0.0		
Nasopharynx	6	5.1	0	0,0		
**Total**	**117**	**100.0**	**4**	**3.4 (0.9-8.5)**		

HNC: Head and neck cancer.

## Discussion

To our knowledge, this is the first study to explore the presence of viral DNA and cellular protein expression in head and neck tumors in West Africa. HPV DNA was detected in 3.4% (95% CI = 0.9%-8.5%) of studied cases but further analyses testing for p16^INK4a^ expression suggested that HPV was not involved in the oncogenic process of the tumors. Expression of p16^INK4a^ is strongly seen in HPV-associated tumors but nearly absent in HPV-negative carcinomas [4] and increases in cases where HPV is oncogenically involved due to the interaction of the viral oncoprotein E7 with pRb [5].

Similar low HPV prevalences have been reported in the African-American community in the USA. In a prospective study conducted by Settle et al. [6], overall HPV positivity was 4% in black patients diagnosed with oropharyngeal cancer versus 34% in white patients. Weinberger et al. [7] found 0% HPV-active HNC in black patients compared to 21% in white patients after immunohistochemical testing with p16^INK4a^. Both studies agree on significant ethnic and biological disparities in HPV prevalence, after adjusting for clinical and sociodemographic characteristics, and support the validity of our study. Further studies are needed to elucidate the mechanism of HPV clearance in African-American and African populations, which share common ancestral origins.

These findings are very different from what has been reported previously in other regions of the world. In a systematic review by Kreimer et al. [3] compiling data on 60 studies focusing on HNSCC, HPV DNA was detected overall in 26% of cases. Reported site-specific HPV prevalence was 23.5% (95% CI = 21.9-25.1%) in oral SCC, 35.6% (95% CI = 32.6-38.7%) in oropharyngeal SCC and 24.0% (95% CI = 21.8-26.3%) in laryngeal SCC [3]. The highest detection rates were found in Asia, followed by the USA and Nordic countries [3].

Low HPV prevalence in invasive HNC in Senegal suggest that other established risk factors such as alcohol and tobacco consumption may play a more significant etiological role than HPV infection in HNC. However, the prevalence of tobacco use in Senegal is relatively low with estimates of 19.9% in men and 1.3% in women [8]. Alcohol consumption is also negligible, especially in women, since Senegal is a predominantly Muslim country with 95% of the population practicing this religion. Thus, the magnitude of the implication of these two classical risk factors in the development of HNC is probably small given the social norms. Therefore, we hypothesize that environmental factors and eating habits may contribute more importantly to the development of HNC.

For example, indoor air pollution causes 6,300 deaths each year in Senegal due to daily exposure to smoke from open burning of wood and charcoal in homes [9]. More than 80% of the households use either wood or charcoal as cooking fuels in peri-urban and rural Senegal. Moreover, burning incense to deodorize and heat the indoor is a widely practiced cultural habit. The aforementioned are potential risk factors for laryngeal and nasopharyngeal cancers as they expose individuals to smoke and dangerous emissions of particles such as CO_2_ and CO [9]. According to the WHO, 3.7% of the burden of disease in developing countries can be attributed to indoor air pollution [9].

Another potential risk factor could be the consumption of “ataya”, a strong and bitter hot tea comparable to “yerba mate” in South America, which has been associated with an increased risk of developing cancer of the oral cavity, larynx and esophagus [10]. On average, at least three rounds of “ataya” are served twice a day in Senegal. The fact that this tea is drank at very hot temperature and consumed with slurps can contribute to an increased risk of cancer.

Poor oral hygiene is another potential risk factor that has been documented [11] and could play a non-negligible role in Senegal. A study in 330 Senegalese university students has shown poor dental health and the need to improve prevention programs [12]. This study was conducted in an educated cohort, which suggests that dental issues may be worse in uneducated, low socio-economic populations with limited access to dental care. Moreover, chewing of kola nuts (Cola acuminate) has also been reported to increase carcinogenesis potential of tobacco in smokers in Nigeria by promoting palatal mucosa keratinization [13]. Consumption of kola nuts is also widespread in Senegal.

Occupational exposure such as jobs in the construction, metal, textile, ceramic, logging, and food industries has been associated with the development of laryngeal cancer [14]. In Senegal, protective masks and safety rules are not applied, as workplace safety regulations are not reinforced mainly due to the fact that the informal sector provides most of the jobs. This reality puts workers in a difficult predicament whereby they expose themselves to hazardous materials.

Our study has some methodological limitations. At sample collection stage, some cases were identified eligible for the study but corresponding blocks were not found as they were sent to laboratories abroad for histopathological evaluation. Additionally, several archival records were lost in one of the main laboratories due to a fire. However, these issues affected all cases regardless of patient characteristics or diagnosis. Another major limitation is the lack of individual data on risk factors: no information on patients besides their gender, age and pathological information were available in the registries. Finally, the small sample size (n = 5) for oropharyngeal tumors may have affected our positivity rate. It is recognized that the highest associations of HPV in HNC have been shown in the oropharynx, particularly in the tonsils, with a positivity of 57% to 82% compared to 0.8% to 9% in other anatomic sites [15].

Our study has several strengths comprising the combined use of highly sensitive assays for HPV DNA detection and a marker of HPV related transforming process such as p16^INK4a^ expression. Additionally, we evaluated the quality of our specimens by means of cellular tubulin detection and subsequently excluded the HPV DNA negative and tubulin negative cases from the statistical analyses. Lastly, all of the study cases were selected from the main anatomy and pathology laboratories in Senegal. Thus, our cohort is representative of the target population as the vast majority of patients are diagnosed in participant centers.

## Conclusion

In Senegal, HPV prevalence in HNC is very infrequent and HPV infection is not associated with carcinogenesis. Therefore, HPV prophylactic vaccines would not have any impact on HNC incidence. Our findings need to be further validated with supplementary studies that include larger case series and the assessment of region-specific risk factors. Additional data will allow to close the gap of knowledge between Western countries and the sub-Saharan African region and to assist health authorities in implementing public health strategies.

## Competing interests

HT served as a consultant and on advisory boards and received speaker fees and travel assistance from Merck-Frosst Canada and Glaxo Smith Kline Pharmaceuticals, Belgium. LA occasionally received travel grants to attend conferences granted by Merck and Sanofi Pasteur MSD. XC received travel grants for scientific meetings and honorarium for consultancy occasionally granted by GlaxoSmithKline, Merck, Sanofi Pasteur MSD. XB received travel grants to conferences/symposia/meetings and honorarium occasionally granted by GlaxoSmithKline, Merck, Sanofi Pasteur MSD, Roche or Qiagen. SS received travel grants to conferences/symposia/meetings occasionally granted by GlaxoSmithKline, Sanofi Pasteur MSD or Qiagen. Other co-authors have no potential conflict of interest to declare.

## Authors’ contributions

CN collected data, performed data analysis and drafted the manuscript. SJ, XC, FXB and LA initiated and designed the study. LA, HT and SJ guided data analysis, interpreted data and edited the paper. XC and FXB made substantial contributions to the manuscript and contributed to data interpretation. NN, YD and MJD interpreted data and edited the paper. JEK performed the laboratory tests and MA the histopathological evaluation. ST managed and cleaned the database. All authors read and approved the final manuscript.
